# Incomplete recovery of bone strength and trabecular microarchitecture at the distal tibia 1 year after return from long duration spaceflight

**DOI:** 10.1038/s41598-022-13461-1

**Published:** 2022-06-30

**Authors:** Leigh Gabel, Anna-Maria Liphardt, Paul A. Hulme, Martina Heer, Sara R. Zwart, Jean D. Sibonga, Scott M. Smith, Steven K. Boyd

**Affiliations:** 1 Human Performance Laboratory, Faculty of Kinesiology, University of Calgary, Calgary, Canada; 2 McCaig Institute for Bone and Joint Health, University of Calgary, 3280 Hospital Drive NW, Calgary, AB T2N 4Z6 Canada; 3 Department of Internal Medicine, Rheumatology and Immunology, German Centre for Immune Therapy, Friedrich-Alexander-Universität Erlangen-Nürnberg and Universitätsklinikum Erlangen, Erlangen, Germany; 4 IU International University of Applied Sciences Erfurt and Department of Nutrition and Food Science, Nutritional Physiology, University of Bonn, Bonn, Germany; 5 Department of Preventive Medicine and Population Health, University of Texas Medical Branch, Galveston, TX USA; 6 Human Heath and Performance Directorate, NASA Lyndon B. Johnson Space Center, Houston, USA; 7 Department of Radiology, Cumming School of Medicine, University of Calgary, Calgary, Canada

**Keywords:** Bone, Osteoporosis

## Abstract

Determining the extent of bone recovery after prolonged spaceflight is important for understanding risks to astronaut long-term skeletal health. We examined bone strength, density, and microarchitecture in seventeen astronauts (14 males; mean 47 years) using high-resolution peripheral quantitative computed tomography (HR-pQCT; 61 μm). We imaged the tibia and radius before spaceflight, at return to Earth, and after 6- and 12-months recovery and assessed biomarkers of bone turnover and exercise. Twelve months after flight, group median tibia bone strength (F.Load), total, cortical, and trabecular bone mineral density (BMD), trabecular bone volume fraction and thickness remained − 0.9% to − 2.1% reduced compared with pre-flight (p ≤ 0.001). Astronauts on longer missions (> 6-months) had poorer bone recovery. For example, F.Load recovered by 12-months post-flight in astronauts on shorter (< 6-months; − 0.4% median deficit) but not longer (− 3.9%) missions. Similar disparities were noted for total, trabecular, and cortical BMD. Altogether, nine of 17 astronauts did not fully recover tibia total BMD after 12-months. Astronauts with incomplete recovery had higher biomarkers of bone turnover compared with astronauts whose bone recovered. Study findings suggest incomplete recovery of bone strength, density, and trabecular microarchitecture at the weight-bearing tibia, commensurate with a decade or more of terrestrial age-related bone loss.

## Introduction

The detrimental effect of spaceflight on skeletal tissue can be profound^1,2^. Decreases in mechanical loading in microgravity cause substantial loss of bone mineral density (BMD) and strength^1,3^ and the deterioration of trabecular microarchitecture^4^. Biochemical studies of bone turnover highlight altered bone metabolism during spaceflight, such that biomarkers of bone resorption increase during spaceflight, while biomarkers of bone formation lag, resulting in net bone loss^4,5^.

Spaceflight-induced bone loss varies between individuals and skeletal sites. For example, bone tissue is better preserved at the non-weight-bearing upper extremities than the weight-bearing lower extremities^3,4^. Recovery of BMD and strength upon return to Earth’s gravity is a lengthy process, and many astronauts’ bones never completely recover^3,6,7^. Determining who is at greatest risk for incomplete recovery of bone tissue is important for understanding feasibility of missions beyond low-Earth orbit.

BMD is traditionally assessed using dual X-ray absorptiometry (DXA); however, DXA is a two-dimensional imaging modality that has limited resolution and cannot differentiate between cortical and trabecular bone compartments. High-resolution peripheral quantitative computed tomography (HR-pQCT), on the other hand, has the highest in vivo imaging resolution currently available (61 μm) to assesses compartmental volumetric BMD, microarchitecture, and estimate bone strength. HR-pQCT predicts fracture risk independent of areal BMD measured by DXA^8^. One study using HR-pQCT in cosmonauts noted persistent deficits in tibia bone strength and total and trabecular BMD 1 year after return from 4- to 6-month space missions^3^. However, the resolution of the first-generation HR-pQCT scanner in that study (82 μm) compared to the new second-generation scanner (61 μm) precluded direct measure of trabecular microarchitecture^9,10^.

The primary aim of this study was to use second-generation HR-pQCT to examine recovery of bone microarchitecture, density, and strength after long-duration spaceflight. Secondary aims included examining the effect of mission duration and exercise on bone recovery; relationships between biochemical measures of bone turnover and bone recovery; and recovery of areal BMD, measured by DXA.

## Results

Study participants included 14 male and three female astronauts with a mean (SD) age at launch of 46.9 (6.7) years, height 177.7 (6.0) cm, and body mass 79.1 (7.7) kg. Missions ranged from 4-to-7-months duration (mean 170 days), with eight astronauts on missions longer than 6-months duration (mean 6.5) and nine astronauts on missions less than 6-months duration (mean 4.9). The mission studied was the first long-duration (> 3 months) flight for 14 astronauts.

### Bone microarchitecture, density, and strength by HR-pQCT

**Figure 1:**
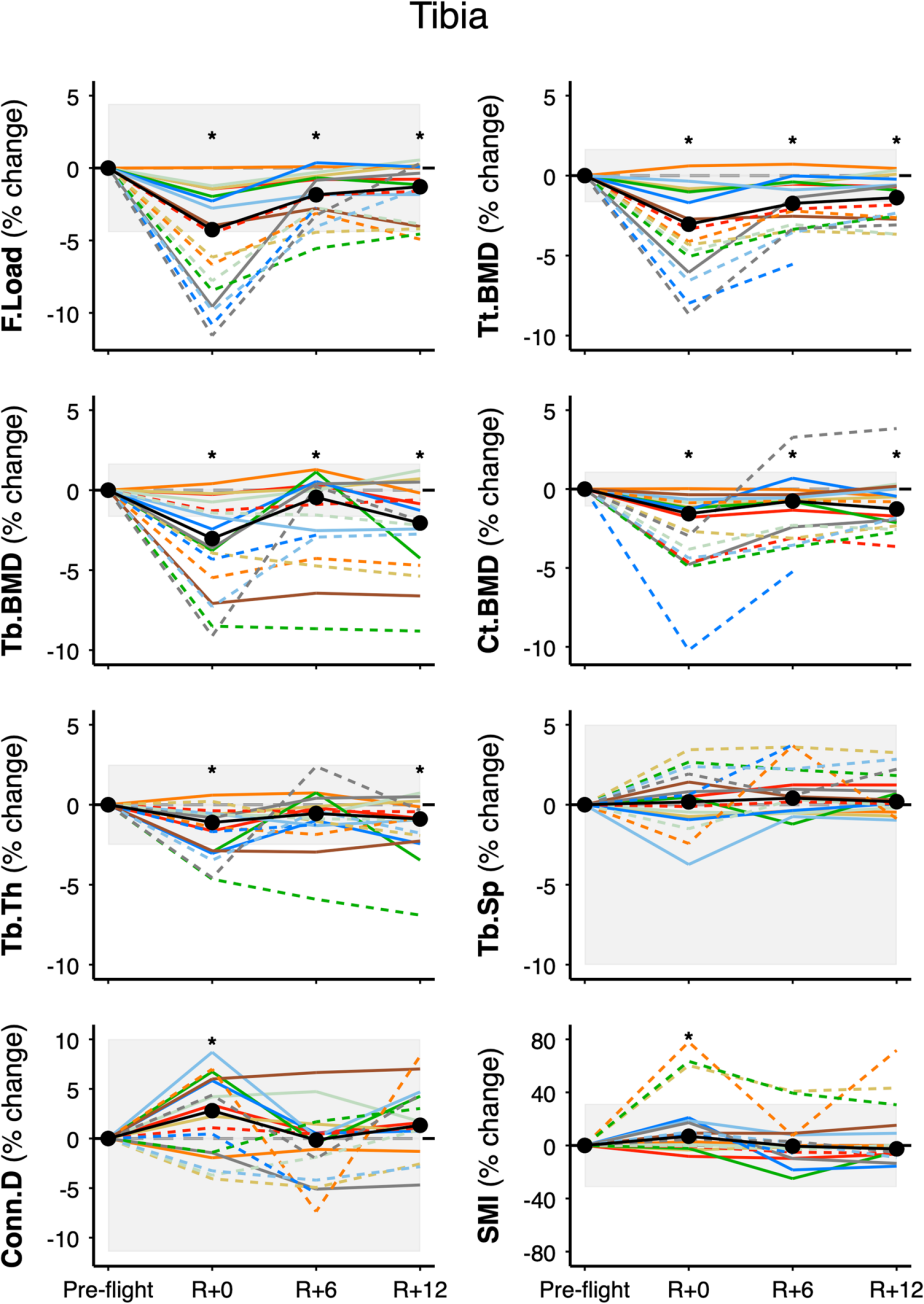
Tibia bone strength, density, and trabecular microarchitecture. Values are percent change from pre-flight at return (R + 0), 6-months (R + 6), and 12-months (R + 12) recovery. Dashed lines for astronauts on > 6-month missions (n = 8) and solid lines for astronauts on < 6-month missions (n = 9). Black circles connected by thick solid line indicate median group change. Shaded bars indicate least significant change^15^. *F.Load* failure load, *Tt.BMD* total bone mineral density, *Ct.BMD* cortical bone mineral density, *Tb.BMD* trabecular bone mineral density, *Tb.Th* trabecular thickness, *Tb.Sp* trabecular separation, *Conn.D* connectivity density, *SMI* structure model index. One astronaut on a > 6-month mission did not complete the R + 12 measure (dashed, blue line). *p < 0.05 from pre-flight. Tb.Th *p* = 0.06 at R + 6.

After 12 months of recovery, tibia failure load (F.Load), total BMD (Tt.BMD), trabecular BMD (Tb.BMD), trabecular bone volume fraction (Tb.BV/TV), trabecular thickness (Tb.Th), and cortical BMD (Ct.BMD) did not recover, remaining below pre-flight values (group median − 0.9% to − 2.1%; Table 1, Fig. 1). Mean connectivity density (Conn.D) and structure model index (SMI) were elevated immediately post-flight but returned to pre-flight values during recovery, suggesting on average that perforations in plate-like trabeculae during spaceflight recovered after return to Earth. At the distal radius, group bone strength, density, and microarchitecture did not differ from pre-flight, nor did radius parameters change in the first year after spaceflight (Table 2, Supplementary Fig. S1).

**Table 1:** Pre-flight HR-pQCT bone variables and absolute and percent change from pre-flight at the distal tibia.

Tibia	Pre-flight	Δ R + 0m	Δ R + 6m	Δ R + 12m
F.Load (N)	10,579.0 (9743.3, 12,159.0)	− **495.0 (**− **950.1, **− **273.0)**^**b**^	− **204.0 (**− **284.7, **− **89.0)**^**b**^	− **152.0 (**− **340.6, 5.1)**^**b**^
		− 4.5% (− 8.5, − 2.0)	− 1.9% (− 3.1, − 0.7)	− 1.3% (− 3.9, 0.0)
Tt.BMD (mg/cm^3^)	326.8 (306.1, 364.1)	− **10.3 (**− **18.6, **− **3.5)**^**b**^	− **6.4 (**− **8.7, **− **1.5)**^**b**^	− **4.5 (**− **9.1, **− **1.4)**^**b**^
		− 3.4% (− 5.1, − 1.0)	− 2.1% (− 3.3, − 0.5)	− 1.4% (− 2.7, − 0.4)
Tb.BMD (mg/cm^3^)	191.3 (178.4, 209.5)	− **6.3 (**− **12.2, **− **2.1)**^**b**^	− **1.7 (**− **5.6, 0.6)**^**b**^	− **3.3 (**− **10.4, **− **0.8)**^**b**^
		− 3.6% (− 5.5, − 1.3)	− 0.9% (− 2.9, 0.3)	− 2.1% (− 4.5, − 0.4)
Tb.BV/TV (%)	27.59 (25.92, 30.12)	− **0.74 (**− **1.33, **− **0.20)**^**b**^	− **0.16 (**− **0.82, 0.08)**^**b**^	− **0.37 (**− **1.16, 0.01)**^**b**^
		− 2.7% (− 4.1, − 0.8)	− 0.6% (− 3, 0.3)	− 1.3% (− 3.5, 0.0)
Tb.Th (mm)	0.274 (0.257, 0.289)	− **0.004 (**− **0.009, **− **0.002)**^**b**^	− 0.002 (− 0.004, 0.000)^**c**^	− **0.002 (**− **0.006, **− **0.001)**^**b**^
		− 1.4% (− 3.0, − 0.6)	− 0.7% (− 1.2, − 0.1)	− 0.9% (− 2.1, − 0.2)
Tb.N (1/mm)	1.38 (1.21, 1.5)	0.00 (− 0.02, 0.01)	− 0.01 (− 0.03, 0.01)	0.00 (− 0.02, 0.01)
		− 0.2% (− 1.1, 1.1)	− 0.4% (− 2.2, 0.7)	0.0% (− 1.8, 0.6)
Tb.Sp (mm)	0.664 (0.638, 0.761)	0.003 (− 0.005, 0.009)	0.003 (− 0.002, 0.013)	0.001 (− 0.002, 0.009)
		0.5% (− 0.8, 1.4)	0.5% (− 0.4, 2.2)	0.2% (− 0.3, 1.5)
Conn.D (1/mm^3^)	2.71 (2.10, 2.98)	**0.06 (**− **0.04, 0.14)**^**a**^	− 0.01 (− 0.09, 0.02)	0.04 (− 0.01, 0.09)
		2.2% (− 1.4, 5.8)	− 0.5% (− 4.2, 0.5)	1.3% (− 0.3, 4.3)
SMI	1.29 (0.66, 1.60)	**0.12 (0.00, 0.19)**^**b**^	0.00 (− 0.06, 0.08)	− 0.02 (− 0.08, 0.14)
		9.3% (− 0.2, 18.7)	− 1.0% (− 6.0, 7.3)	− 2.5% (− 6.2, 12.2)
Ct.BMD (mg/cm^3^)	927.3 (898.5, 955.5)	− **17.3 (**− **39.7, **− **8.3)**^**b**^	− **7.2 (**− **27.1, **− **5.3)**^**b**^	− **12.0 (**− **20.9, **− **2.0)**^**b**^
		− 1.8% (− 4.4, − 0.9)	− 0.8% (− 3.1, − 0.6)	− 1.3% (− 2.2, − 0.2)
Ct.Th (mm)	1.59 (1.45, 1.67)	− 0.01 (− 0.04, 0.02)	0.00 (− 0.03, 0.01)	0.01 (0.00, 0.03)
		− 0.8% (− 2.3, 1.4)	− 0.2% (− 1.7, 0.8)	0.5% (− 0.2, 2.1)
Ct.Po (%)	2.46 (2.12, 3.31)	0.20 (0.01, 0.43)	0.18 (0.08, 0.26)	0.19 (0.08, 0.56)
		7.5% (1.1, 16.1)	7.9% (2.4, 13.7)	9.2% (4.4, 15.9)

Data are median (IQR) for pre-flight, absolute median change, and pairwise percent median change from pre-flight. n = 17. ^**a**^***p*** **<** **0.05**; ^**b**^***p*** **<** **0.01** vs. pre-flight based on linear mixed effects models. ^**c**^***p*** **=** **0.06**. *R* + *0m* return, *R* + *6m* 6 months after return, *R* + *12m* 12 months after return, *F.Load* failure load, *Tt.BMD* total bone mineral density, *Tb.BMD* trabecular BMD, *Tb.BV/TV* trabecular bone volume fraction, *Tb.Th* trabecular thickness, *Tb.N* trabecular number, *Tb.Sp* trabecular separation, *Ct.BMD* cortical BMD, *Ct.Th* cortical thickness, *Ct.Po* cortical porosity, *Conn.D* connectivity density, *SMI* structure model index.

**Table 2:** Pre-flight HR-pQCT bone variables and absolute and percent change from pre-flight at the distal tibia and radius.

Radius	Pre-flight	Δ R + 0m	Δ R + 6m	Δ R + 12m
F.Load (N)	3977.2 (3595.2, 4792.1)	11.7 (− 60.1, 205.6)	− 38.8 (− 84.4, 56.6)	26.9 (− 57.4, 138.1)
		0.4% (− 1.3, 5.0)	− 0.8% (− 2.2, 1.2)	0.6% (− 1.3, 2.6)
Tt.BMD (mg/cm^3^)	336.6 (312.3, 362.3)	− 0.3 (− 2.6, 2.7)	− 0.3 (− 2.6, 2.6)	0.1 (− 4.0, 2.5)
		− 0.1% (− 0.9, 0.7)	− 0.1% (− 0.8, 0.8)	0.0% (− 1.1, 0.8)
Tb.BMD (mg/cm^3^)	180.5 (162.1, 192.5)	0.5 (− 2.4, 2.5)	0.2 (− 0.8, 1.6)	− 0.2 (− 3.0, 2.1)
		0.3% (− 1.7, 1.3)	0.1% (− 0.5, 0.8)	− 0.1% (− 1.6, 1.1)
Tb.BV/TV (%)	26.35 (23.03, 27.61)	0.05 (− 0.34, 0.22)	0.01 (− 0.33, 0.16)	− 0.12 (− 0.59, 0.35)
		0.2% (− 1.4, 1.0)	0.0% (− 1.2, 0.6)	− 0.4% (− 2.9, 1.3)
Tb.Th (mm)	0.241 (0.233, 0.253)	− 0.002 (− 0.004, − 0.000)	− 0.000 (− 0.001, 0.001)	− 0.001 (− 0.003, 0.002)
		− 0.9% (− 1.7, − 0.1)	− 0.1% (− 0.6, 0.5)	− 0.4% (− 1.3, 0.6)
Tb.N (1/mm)	1.54 (1.40, 1.59)	− 0.01 (− 0.05, 0.00)	− 0.01 (− 0.05, 0.03)	− 0.01 (− 0.08, 0.04)
		− 1.0% (− 3.7, 0.3)	− 0.8% (− 2.9, 1.8)	− 0.9% (− 4.9, 2.8)
Tb.Sp (mm)	0.592 (0.572, 0.653)	0.002 (− 0.001, 0.011)	0.002 (− 0.006, 0.011)	0.000 (− 0.008, 0.017)
		0.3% (− 0.2, 1.8)	0.3% (− 0.9, 1.6)	0.0% (− 1.4, 2.8)
Conn.D (1/mm^3^)	3.24 (2.67, 3.40)	0.00 (− 0.13, 0.08)	− 0.02 (− 0.21, 0.03)	− 0.06 (− 0.24, 0.01)
		− 0.1% (− 4.0, 2.8)	− 0.4% (− 8.0, 1.1)	− 2.0% (− 8.5, 0.5)
SMI	2.34 (1.74, 2.83)	− 0.06 (− 0.45, 0.14)	0.00 (− 0.20, 0.30)	− 0.01 (− 0.42, 0.32)
		− 2.4% (− 14.9, 6.9)	− 0.3% (− 11.9, 12.2)	− 0.6% (− 22.6, 9.2)
Ct.BMD (mg/cm^3^)	916.3 (894.3, 951.1)	1.7 (− 4.0, 10.2)	− 3.2 (− 5.6, 3.2)	0.4 (− 6.2, 7.3)
		0.2% (− 0.4, 1.1)	− 0.4% (− 0.6, 0.3)	0.0% (− 0.7, 0.8)
Ct.Th (mm)	1.14 (1.00, 1.18)	0.00 (− 0.01, 0.02)	0.00 (− 0.01, 0.01)	0.00 (− 0.01, 0.02)
		0.1% (− 0.9, 1.5)	0.2% (− 1.1, 0.8)	0.1% (− 0.7, 1.4)
Ct.Po (%)	0.63 (0.39, 0.77)	0.02 (− 0.09, 0.09)	0.00 (− 0.07, 0.08)	0.04 (− 0.05, 0.17)
		5.1% (− 11.0, 16.0)	0.9% (− 10.5, 21.2)	7.5% (− 11.4, 29.5)

Data are median (IQR) for pre-flight, absolute median change, and pairwise percent median change from pre-flight. n = 16. No differences vs. pre-flight based on linear mixed effects models. *R* + *0m* return, *R* + *6m* 6 months after return, *R* + *12m* 12 months after return, *F.Load* failure load, *Tt.BMD* total bone mineral density, *Tb.BMD* trabecular BMD, *Tb.BV/TV* trabecular bone volume fraction, *Tb.Th* trabecular thickness, *Tb.N* trabecular number, *Tb.Sp* trabecular separation, *Ct.BMD* cortical BMD, *Ct.Th* cortical thickness, *Ct.Po* cortical porosity, *Conn.D* connectivity density, *SMI* structure model index.

### Effect of mission duration

**Figure 2:**
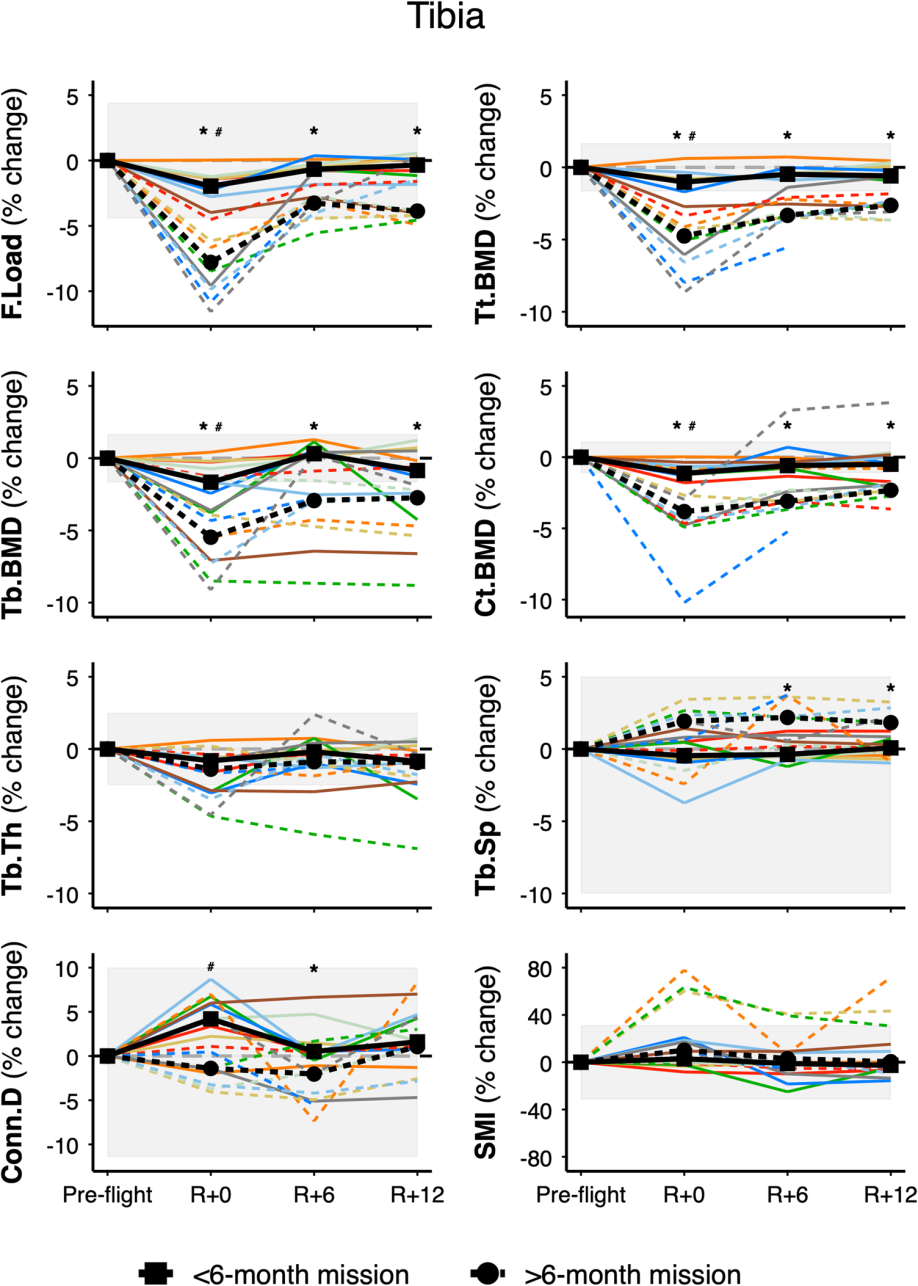
Tibia bone strength, density, and trabecular microarchitecture by mission duration. Values are percent change from pre-flight at return (R + 0), 6-months (R + 6), and 12-months (R + 12) recovery. Dashed lines for astronauts on > 6-month missions (n = 8) and solid lines for astronauts on < 6-month missions (n = 9). Black circles connected by thick dashed line indicate median change for astronauts on > 6-month missions and black squares connected by thick solid line indicate median change for astronauts on < 6-month missions. Shaded bars indicate least significant change^15^. *F.Load* failure load, *Tt.BMD* total bone mineral density, *Ct.BMD* cortical bone mineral density, *Tb.BMD* trabecular bone mineral density, *Tb.Th* trabecular thickness, *Tb.Sp* trabecular separation, *Conn.D* connectivity density, *SMI* structure model index. One astronaut on a > 6-month mission did not complete the R + 12 measure (dashed, blue line). *p < 0.05 from pre-flight for astronauts on > 6-month missions; ^#^*p* < 0.05 from pre-flight for astronauts on < 6-month missions.

We determined the influence of mission duration (< 6 months = nine astronauts vs. > 6 months = eight astronauts) on bone recovery using mixed effects models with a significant time by mission duration interaction term. At the distal tibia, mission duration predicted loss and recovery of F.Load, Tt.BMD, Tb.BMD, Ct.BMD, and trabecular separation (Tb.Sp), indicating greater loss and incomplete recovery in astronauts on longer duration missions (Fig. 2). For astronauts on longer missions, median percent change (and absolute change from mixed model analysis) from pre-flight to 12-months recovery at the tibia for F.Load was − 3.9% (− 333.9 N, 95% CI − 497.5 to − 170.2), for Tt.BMD was − 2.6% (− 9.4 mgHA/cm^3^, − 12.2 to − 6.6), for Tb.BMD was − 2.7% (− 7.7 mgHA/cm^3^, − 11.4 to − 3.9), for Ct.BMD was − 2.3% (− 16.5 mgHA/cm^3^, − 27.2 to − 5.8), and for Tb.Sp was 1.8% (0.010 mm, 0.002 to 0.018). In contrast, for astronauts on shorter missions, median percent change (and absolute change from mixed model analysis) for F.Load was − 0.4% (− 79.9 N, 95% CI − 227.3 to 67.5), for Tt.BMD was − 0.6% (− 1.8 mgHA/cm^3^, − 4.3 to 0.8), for Tb.BMD was − 0.9% (− 3.0 mgHA/cm^3^, − 6.4 to 0.3), for Ct.BMD was − 0.5% (− 6.8 mgHA/cm^3^, − 16.5 to 2.9), and for Tb.Sp was 0.1% (0.001 mm, − 0.006 to 0.008). A similar time by mission duration trend (p = 0.07) was seen for Tb.BV/TV where change was − 2.5% (− 0.85%, − 1.30 to − 0.40) and − 1.1% (− 0.34%, − 0.75 to 0.06) in astronauts on longer and shorter missions, respectively. Mission duration did not predict incomplete recovery of tibia Tb.Th (− 0.9% for long and short duration missions, p = 0.76).

At the radius, bone recovery was influenced by mission duration (significant interaction between mission duration and time) for F.Load, Tt.BMD, Tb.BMD, Tb.BV/TV, Ct.BMD, Conn.D, and SMI (Supplementary Fig. S2). In astronauts on longer missions, persistent losses were observed such that median percent change (and absolute change from mixed model analysis) between pre-flight and 12-month recovery was negative: − 1.4% (− 62.7 N, 95% CI − 187.8 to 62.4) for F.Load, − 1.2% (− 3.1 mgHA/cm^3^, − 4.9 to − 1.4) for Tt.BMD, − 2.4% (− 4.0 mgHA/cm^3^, − 5.9 to − 2.1) for Tb.BMD, − 3.7% (− 0.76%, − 1.09 to − 0.43) for Tb.BV/TV, and − 0.7% (− 10.0 mgHA/cm^3^, − 16.4 to − 3.5) for Ct.BMD. In contrast, in astronauts on shorter missions, persistent gains were observed such that median change at 12-months recovery was positive: 0.9% (159.8 N, 95% CI 49.5 to 270.2) for F.Load, 0.7% (2.4 mgHA/cm^3^, 0.9 to 3.9) for Tt.BMD , 0.8% (1.9 mgHA/cm^3^, 0.2 to 3.5) for Tb.BMD, 1.2% (0.35, 0.06 to 0.65) for Tb.BV/TV, and 0.5% (4.9 mgHA/cm^3^, − 0.8 to 10.6) for Ct.BMD. After 12 months of recovery, Conn.D was − 7.4% (− 0.301/mm^3^, − 0.47 to − 0.12) in astronauts on longer missions and − 0.9% (0.001/mm^3^, − 0.15 to 0.16) in astronauts on shorter missions, while SMI was 11.9% (0.39, 0.12 to 0.66) greater in astronauts on longer missions compared with − 3.5% (− 0.29, − 0.53 to − 0.06) in astronauts on shorter missions.

### Areal BMD by DXA

**Figure 3:**
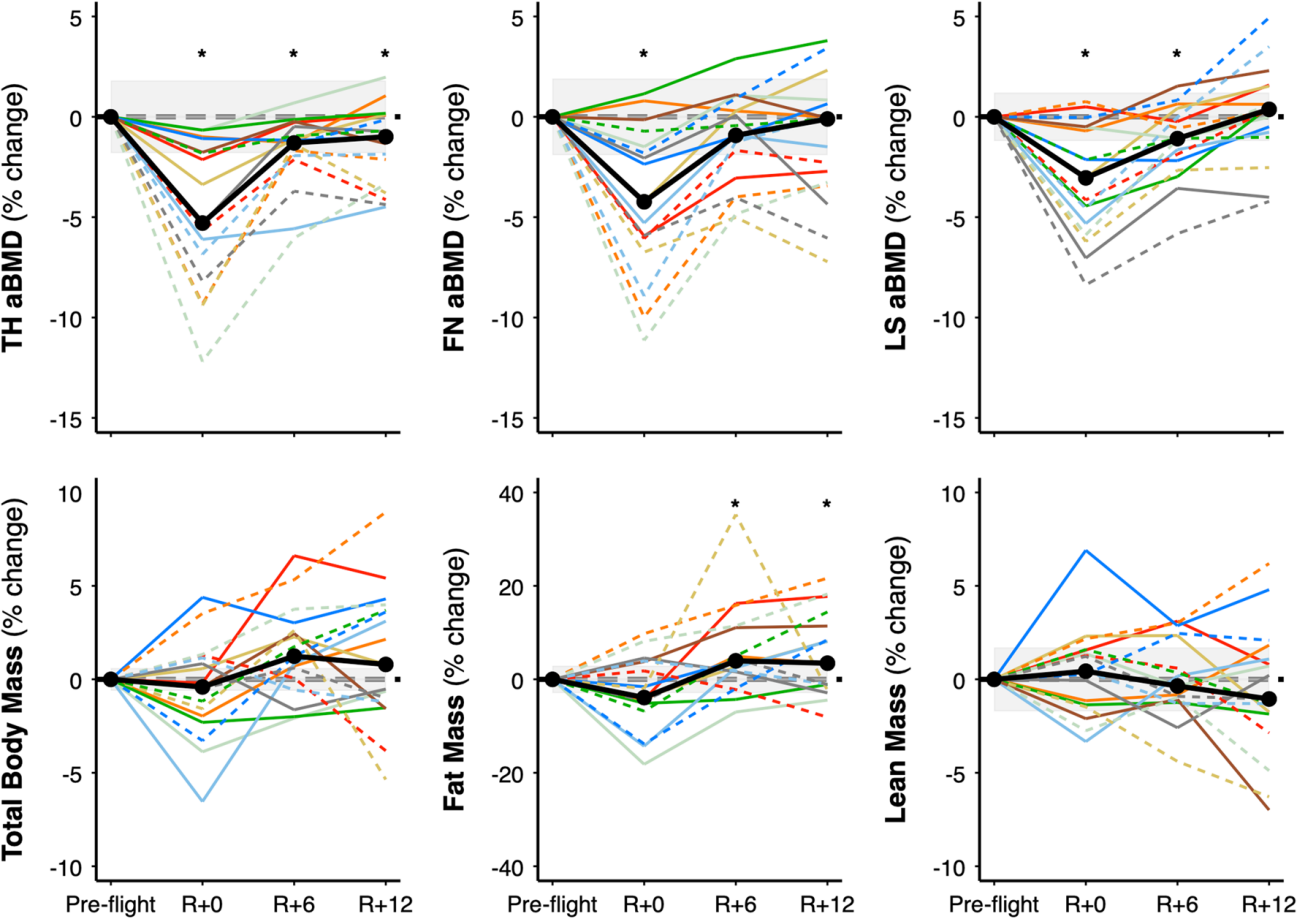
Total hip (TH), femoral neck (FN), and lumbar spine (LS) aBMD, total body mass, total body fat mass, and total body lean mass less bone mineral content. Values are percent change from pre-flight at return (R + 0), 6-months (R + 6), and 12-months (R + 12) recovery. Dashed lines for astronauts on > 6-month missions (n = 8) and solid lines for astronauts on < 6-month missions (n = 9). Black circles connected by thick solid line indicates median group change. Shaded error bars indicate least significant change^15^. *p < 0.05 from pre-flight.

Femoral neck and lumbar spine areal BMD (aBMD) recovered by 6 and 12 months after return from space, respectively, while total hip aBMD remained 1% lower compared with pre-flight (Table 3, Fig. 3). Total body mass, lean mass, and fat mass did not differ immediately after spaceflight; however, at 6- and 12-months recovery fat mass and percent body fat were elevated and percent lean mass was reduced (Table 3, Fig. 3). As with HR-pQCT measures, mission duration significantly predicted recovery of several DXA variables (Fig. 3). At the total hip and femoral neck, aBMD recovery was incomplete in astronauts on longer (> 6 months) vs. shorter (< 6 months) duration missions. Specifically, after 12 months of recovery, median aBMD was − 2.7% at the total hip and − 2.8% at the femoral neck in astronauts on longer missions compared with 0.08% and − 0.01%, respectively, in astronauts on shorter missions.

**Table 3:** Pre-flight DXA bone variables and absolute and percent change from pre-flight.

	Pre-flight	Δ R + 0m	Δ R + 6m	Δ R + 12m
**Bone densitometry**				
FN aBMD (g/cm^2^)	0.835 (0.803, 0.902)	− **0.041 (**− **0.054, **− **0.013)**^**b**^	− 0.007 (− 0.03, 0.003)	− 0.001 (− 0.028, 0.006)
		− 4.2% (− 6.1, − 1.5)	− 0.9% (− 3.1, 0.3)	− 0.1% (− 3.3, 0.6)
TH aBMD (g/cm^2^)	1.026 (0.982, 1.113)	− **0.052 (**− **0.065, **− **0.018)**^**b**^	− **0.013 (**− **0.019, **− **0.005)**^**b**^	− **0.011 (**− **0.035, 0.001)**^**a**^
		− 5.3% (− 6.8, − 1.8)	− 1.3% (− 1.9, − 0.5)	− 1.0% (− 3.3, 0.1)
LS aBMD (g/cm^2^)	1.072 (1.017, 1.143)	− **0.035 (**− **0.053, **− **0.006)**^**b**^	− **0.012 (**− **0.025, 0.000)**^**a**^	0.004 (− 0.007, 0.017)
		− 3.0% (− 5.3, − 0.5)	− 1.1% (− 2.2, 0.0)	0.4% (− 0.8, 1.5)
**Body mass and composition**				
Total mass (g)	79,663.9 (75,413.5, 86,955.1)	− 278.6 (− 1362.3, 972.1)	1011.1 (53.2, 2025.7)	710.8 (− 1025.8, 2937.5)
		− 0.4% (− 2.0, 1.2)	1.2% (0.1, 2.6)	0.8% (− 1.2, 3.7)
Total fat mass (g)	20,106.4 (17,442.3, 24,526.7)	− 759.8 (− 1289.6, 747.7)	**798.9 (277.4, 2183.7)**^**a**^	**695.1 (**− **275.5, 2251.5)**^**b**^
		− 3.9% (− 5.1, 3.8)	4.0% (1.6, 11.1)	3.5% (− 1.1, 11.4)
Total lean mass (g)^c^	56,544.5 (50,906.9, 62,328.6)	272.4 (− 811.1, 802.9)	− 181.4 (− 583.4, 1439.8)	− 657.5 (− 1216.7, 585.8)
		0.4% (− 1.4, 1.6)	− 0.4% (− 1.1, 2.3)	− 1.0% (− 1.9, 1.1)
Total fat (%)	25.9 (23.6, 27.5)	− 0.8 (− 1.5, 0.6)	**0.7 (0.1, 1.8)**^**a**^	**0.6 (0.0, 2.2)**^**b**^
		− 2.9% (− 5.7, 2.7)	3.2% (0.4, 7.4)	3.2% (− 0.1, 10.3)
Total lean (%)^a^	70.3 (69.1, 73.3)	0.7 (− 0.5, 1.7)	− **0.7 (**− **1.7, 0.1)**^**a**^	− **0.8 (**− **2.0, 0.0)**^**b**^
		1.0% (− 0.7, 2.4)	− 1.0% (− 2.2, 0.1)	− 1.0% (− 2.5, 0.0)

Data are median (IQR) for pre-flight, absolute median change, and pairwise percent median change from pre-flight.

^**a**^***p*** **<** **0.05**; ^**b**^***p*** **<** **0.01** for difference vs. pre-flight based on linear mixed effects models.

^c^Total lean mass less bone mineral content. *LS* lumbar spine, *FN* femoral neck, *TH* total hip, *aBMD* areal bone mineral density.

### HR-pQCT versus DXA

Incomplete recovery of tibia Tt.BMD by HR-pQCT was seen in nine of 17 astronauts (Fig. 1; deficits larger than the least significant change (LSC) of 1.7%). All eight astronauts on missions greater than 6-months duration exceeded the LSC for tibia Tt.BMD at R + 12 (Fig. 2). In contrast, incomplete recovery of total hip aBMD by DXA was present in seven of 17 astronauts (Fig. 3; deficits larger than the LSC of 1.8%). Six of eight astronauts on missions greater than 6-months duration demonstrated losses greater than the LSC for total hip aBMD. Of two astronauts on longer missions whose total hip aBMD recovered, one did not complete the corresponding HR-pQCT scan; thus, we do not have comparison data at 12-month follow-up. The other astronaut whose total hip aBMD by DXA recovered demonstrated large persistent deficits by HR-pQCT: − 3% in Tt.BMD, − 9% in Tb.BMD, − 7% in Tb.Th, and − 5% in F.Load (see dashed, emerald-green line in Figs. 1 and 3).

### Biomarkers of bone turnover

**Figure 4:**
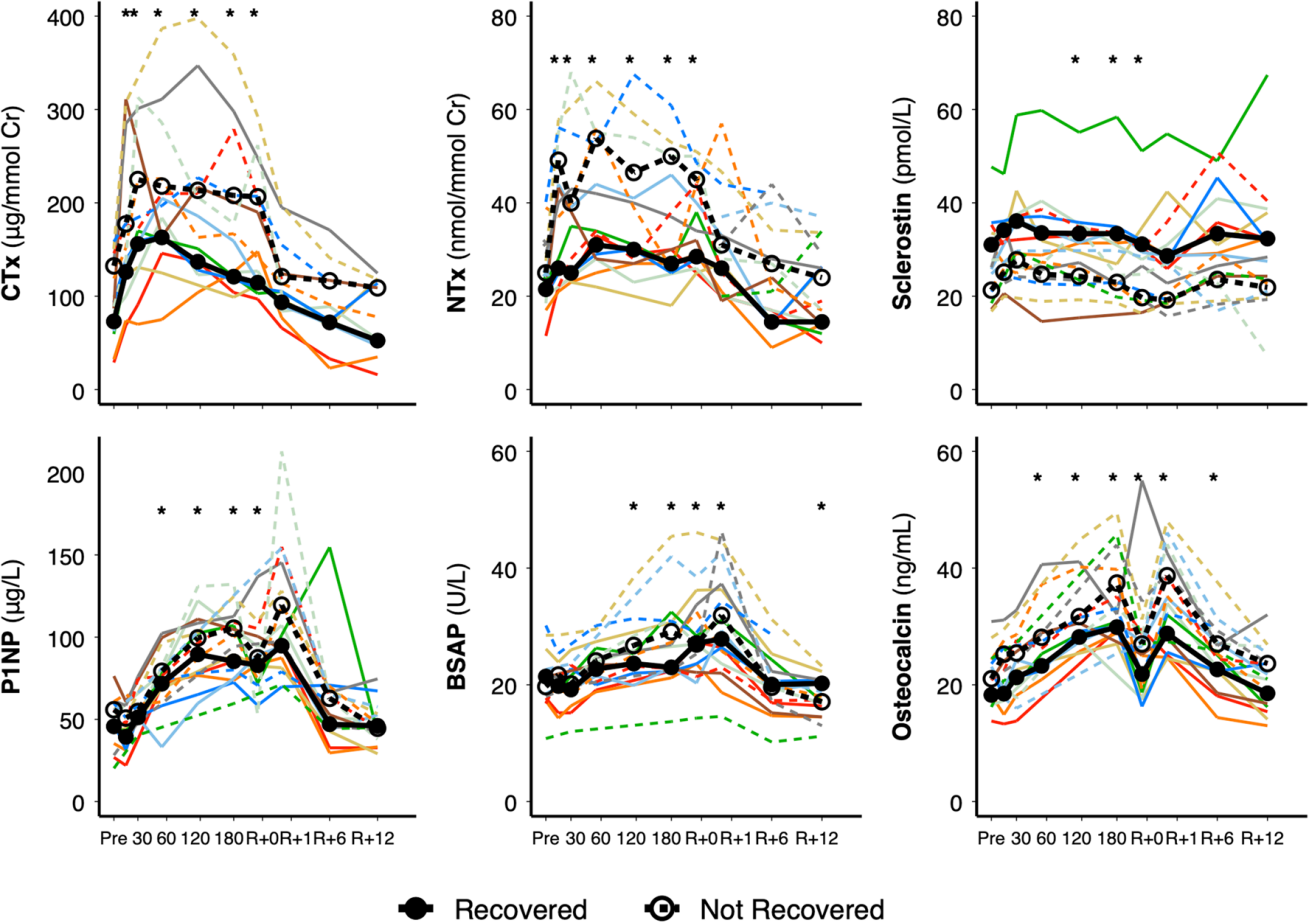
Biomarkers of bone turnover relative to bone recovery at pre-flight (Pre), flight days 15, 30, 60, 120, 180, and return (R + 0), 1-month (R + 1), 6-months (R + 6), and 12-months (R + 12) recovery. Median group change for astronauts who recovered (n = 8) total BMD at R + 12 are indicated by black circles connected by a thick solid line, while median group change in astronauts who did not recover (n = 9) total BMD at R + 12 are indicated by open circles connected by a thick dashed line. Colored dashed lines for astronauts on > 6-month mission and solid lines for astronauts on < 6-month mission. *CTx* type I collagen C-terminal cross-linked telopeptide, *NTx* type I collagen N-terminal cross-linked telopeptide, Sclerostin, *P1NP* procollagen type 1 amino-terminal propeptide, *BSAP* bone-specific alkaline phosphatase, Osteocalcin. Note: three astronauts on > 6-month missions did not complete CTx measures or in-flight NTX measures, and missed in-flight day 15, 60, and 120 measures for sclerostin, P1NP, BSAP, and osteocalcin. Thus, for NTx, these astronauts are indicated by an ‘x’ at pre-flight and trajectory between R + 0 and R + 12. *p < 0.05 from pre-flight for entire group. CTx, NTx, sclerostin, and osteocalcin were significantly different (*p* < 0.05) at all time points between astronauts who recovered tibia total BMD and astronauts whose tibia total BMD did not recover.

Elevated in-flight urinary biomarkers of bone resorption (CTx, type I collagen C-terminal cross-linked telopeptide; NTx, type I collagen N-terminal cross-linked telopeptide) returned to pre-flight values by 1 month after spaceflight (Fig. 4, Supplementary Table S1). Serum biomarkers of bone formation (BSAP, bone-specific alkaline phosphatase and P1NP, procollagen type 1 amino-terminal propeptide) returned to pre-flight values by R + 6 months. BSAP demonstrated further decline such that it was significantly lower at R + 12 months compared with before spaceflight. Osteocalcin, a serum biomarker of bone turnover, remained elevated until R + 6 months but returned to pre-flight by R + 12 months. Serum calcium was reduced at R + 1 month and PTH was elevated at R + 1 and R + 6 months compared with pre-flight (Supplementary Fig. S3, Supplementary Table S1). Receptor activator of nuclear factor kappa-Β ligand (RANKL) was elevated at R + 6 months.

We explored whether bone metabolism differed between astronauts who recovered (n = 8) or did not recover (n = 9) tibia Tt.BMD (Fig. 4). There were significant main effects of recovery-group for CTx and NTx (expressed per creatinine excretion for both), osteocalcin, and sclerostin. Main effects indicated that CTx, NTx, and osteocalcin were lower, while sclerostin was higher, at all times in astronauts whose bone recovered compared with astronauts who did not recover. Before spaceflight, CTx was 74 ± 13 vs. 125 ± 13 μg/mmolCr, NTx was 21 ± 2 vs. 28 ± 3 nmol/mmolCr, osteocalcin was 20.0 ± 1.9 vs. 22.2 ± 1.1 ng/mL, and sclerostin was 32.6 ± 2.5 vs. 22.7 ± 2.0 pmol/L, respectively, in astronauts whose bone recovered compared with astronauts who did not recover. Significant recovery-group by time interactions indicated greater osteoprotegerin (OPG) at FD30 and landing (R + 0) followed by reduced OPG at R + 12 in astronauts whose bone recovered compared to those who did not recover (Supplementary Fig. S3, Supplementary Table S1). Urinary creatinine excretion was elevated while phosphorus was reduced at FD120 and FD180 in astronauts who recovered compared to those who did not recover.

### Exercise and bone recovery

**Figure 5:**
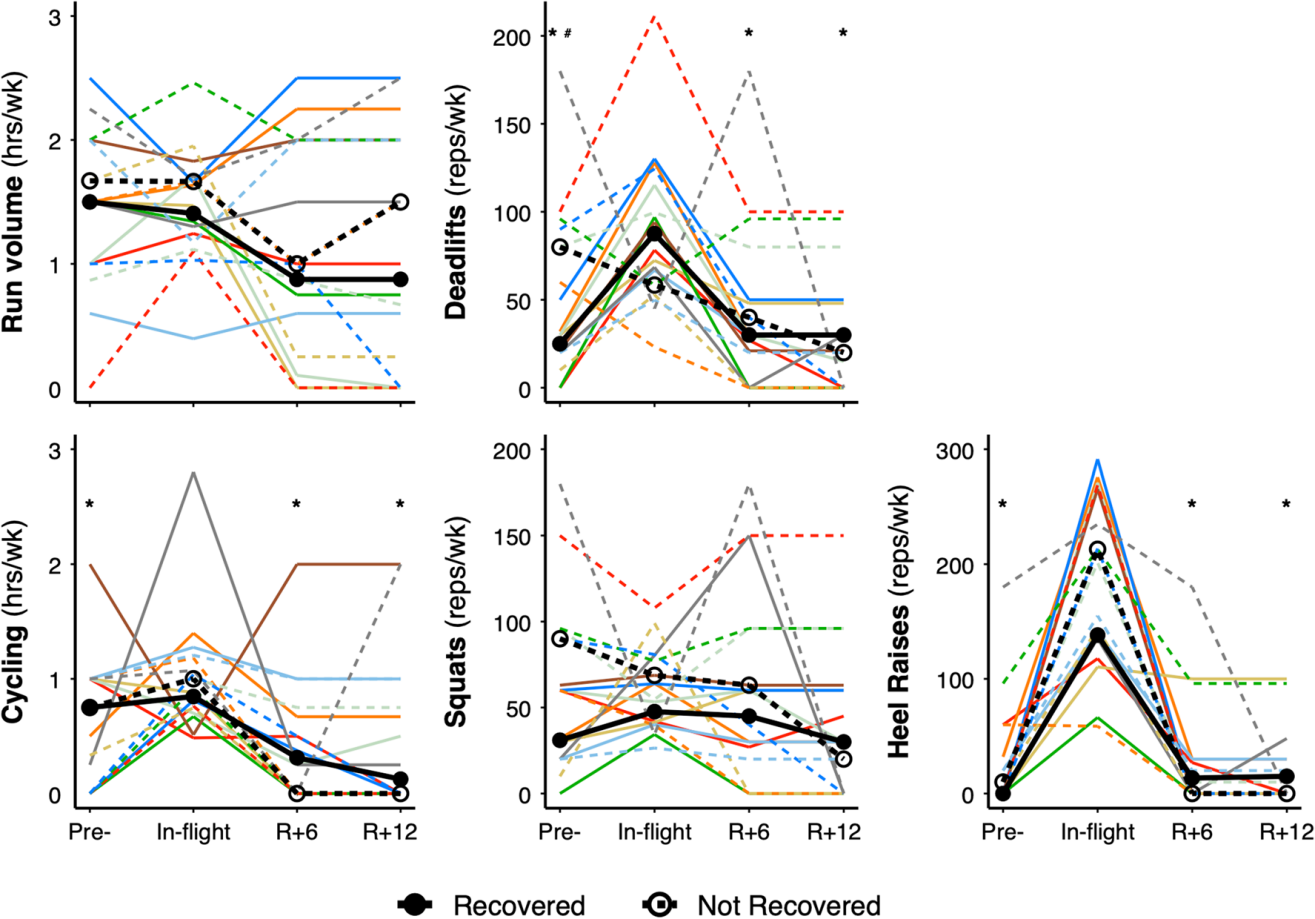
Exercise volume relative to bone recovery pre-flight, in-flight, 6-months (R + 6) and 12-months (R + 12) recovery. Median group change for astronauts who recovered (n = 8) total BMD at R + 12 are indicated by black circles connected by a thick line, while median group change in crewmembers who did not recover (n = 9) total BMD at R + 12 are indicated by open circles connected by a thick dashed line. Colored dashed lines for astronauts on > 6-month mission and solid lines for astronauts on < 6-month mission. *p < 0.05 from in-flight for entire group. ^#^*p* < 0.05 between astronauts whose tibia total BMD recovered and astronauts whose tibia total BMD did not recover.

Figure 5 depicts change in exercise volume (hours per week) across time for running and cycling and change in repetitions per week for deadlifts, squats, and heel raises. Running volume did not significantly differ across time, although astronauts tended to run less after spaceflight compared to in-flight (95% CI − 0.621 to 0.003; p = 0.09). Cycling volume was lower before and after spaceflight compared with in-flight (− 0.93 to − 0.06; p = 0.003). Astronauts performed fewer repetitions per week of deadlifts (− 82 to − 16; p < 0.001) and heel raises (− 201 to − 123; p < 0.001) before and after spaceflight compared with in-flight. Repetitions per week of squats did not differ across time (− 42 to 27; p = 0.19).

Astronauts whose tibia Tt.BMD recovered after spaceflight demonstrated significantly greater increases in in-flight deadlift volume (in-flight repetitions vs. pre-flight repetitions) compared with astronauts who did not recover Tt.BMD (p = 0.05 for time by recovery-group interaction; Fig. 5). In astronauts who recovered Tt.BMD, median deadlift volume increased from pre- to in-flight by 63 repetitions per week (25 reps/wk pre-flight to 88 reps/wk in-flight), while median deadlift volume decreased by 22 repetitions per week (80 to 58 reps/wk) in astronauts who did not recover Tt.BMD. Neither running, cycling, squats, nor heel raise volume were associated with bone recovery.

## Discussion

One year after returning from long-duration spaceflight, most astronauts demonstrated incomplete recovery of bone density, strength, and trabecular thickness at the weight-bearing distal tibia. Notably, incomplete recovery of bone density and strength was more pronounced in astronauts who flew on longer duration missions, for whom bone loss after spaceflight was substantially greater than astronauts on shorter missions^4^. The relevance of mission duration for skeletal health is increasingly important as longer missions to the ISS become more frequent and exploration-class missions are being planned for near future.

Because of its enhanced surface area, trabecular bone is more actively remodeled than cortical bone^11^. Although bone may recover on the surfaces of existing trabeculae, if trabeculae thin to the extent of dissociating from one another (e.g., perforations in rod-like trabeculae) they are unlikely to re-connect when reloaded^12^. Thus, the ability to recover trabecular microarchitecture after prolonged unloading may differ depending on whether loss is localized to plate- versus rod-like trabeculae. Most astronauts with perforations in rod-like trabeculae at the tibia (reductions in Conn.D; Fig. 1) did not restore pre-flight connectivity. We speculate that perforation of trabecular rods results in permanent dissociation of trabeculae and irreversible loss of rod-like trabecular structures. Conversely, astronauts with perforations in plate-like trabeculae (increased Conn.D) were able to recover pre-flight connectivity. Although prior investigations used a range of imaging modalities to demonstrate impaired recovery of bone density and strength in weight-bearing regions after spaceflight, including HR-pQCT (first-generation)^3^, pQCT^13^, QCT^14,15^, and DXA^6^, earlier investigations were unable to directly measure changes in trabecular microarchitecture. The resolution of second-generation HR-pQCT allowed us to directly assess changes in trabecular microarchitecture and identify trabecular thinning and changes in connectivity.

Long-term follow-up of astronauts beyond 1 year is needed to clarify the clinical significance of non-recoverable trabecular thickness and connectivity, and to determine fracture risk. Permanent damage to the skeleton induced by spaceflight may lead to earlier-onset osteoporosis and fragility, particularly when paired with normal age-related declines^16^. DXA scans are routine for monitoring astronauts’ skeletal health; however, DXA cannot distinguish between bone compartments, nor can it measure microarchitecture. Thus, DXA may incorrectly identify bone as recovered if changes in trabecular microarchitecture are decoupled from total bone density (e.g., incomplete recovery in the trabecular region could be obscured by recovery of cortical BMD). Overall patterns of bone recovery were analogous between the distal tibia measured by HR-pQCT and the total hip measured by DXA, indicating skeletal sites that are weight-bearing on Earth adapt similarly to unloading in space. However, we found striking differences between HR-pQCT and DXA for monitoring individual bone recovery. Astronauts who experienced substantial trabecular bone loss and incomplete recovery of trabecular BMD by HR-pQCT were incorrectly identified by DXA as having recovered BMD. To illustrate, DXA identified aBMD as recovered in one astronaut who presented with the largest persistent deficits in HR-pQCT-measured trabecular BMD, thickness, and bone strength. Because aBMD by DXA recovered, this astronaut (and astronauts on prior missions with similar undetected compromised trabecular bone structure and strength) would probably not be referred to a specialist for further monitoring or treatment. These findings support including imaging modalities capable of assessing trabecular microarchitecture (i.e., HR-pQCT) in skeletal surveillance programs.

Although bone partially recovered at the tibia in all astronauts after 1-year, sustained deficits in total BMD were comparable to bone loss after non-weight-bearing due to an anterior cruciate ligament injury or osteotomy^17^, and exceed losses sustained after a tibial bone stress injury^18^. Given such precipitous (and in most cases, persistent) loss due to weightlessness, spaceflight-induced bone loss is likened to age-related bone loss. However, patterns of spaceflight bone loss differ from age-related bone loss. For example, age-related declines in tibia cortical BMD exceed that of trabecular BMD^16^, while spaceflight induces the opposite adaptation—substantially greater declines in the trabecular bone compartment. Additionally, we did not observe the cortical thinning or increases in cortical porosity that typifies normal aging. Thus, spaceflight causes reductions in bone strength and total BMD akin to several decades of age-related bone loss, but how and where bone is lost differs from aging on Earth. Monitoring these compartmental differences in response to spaceflight is only possible using 3D technologies such as HR-pQCT or QCT in general.

Unlike the weight-bearing tibia, we did not observe global post-flight changes at the distal radius. Our findings contrast stark declines in radius total and trabecular BMD reported during post-flight recovery in 13 cosmonauts following 4-to-6-month space missions in the Early Detection of Osteoporosis in Space study^3^. Although we did not observe the same group-level declines in BMD as Vico and colleagues^3^, astronauts on longer missions demonstrated persistent loss of BMD and strength after spaceflight and a few experienced declines in BMD during post-flight recovery. We suspect bone loss at the radius reflects relative unloading after return to Earth, as the upper limbs may experience increased loading on-orbit for manoeuvring around the space station.

Identifying factors affecting skeletal recovery provides insight into optimizing strategies to mitigate loss and enhance recovery of the skeleton. Incomplete recovery of bone density and strength at the tibia was more likely in astronauts who incurred the greatest losses during spaceflight; thus, identifying preventative measures and improving in-flight countermeasures is imperative. In the weightless space environment, it is challenging to impart loads on the body akin to those experienced on Earth. The greatest loads sustained by the lower body on-orbit occur during treadmill running and resistance training^19^. Our previous analysis noted the importance of change in exercise volume for preventing spaceflight bone loss, such that astronauts were more likely to preserve their bone density and strength if they increased in-flight lower body resistance training volume relative to pre-flight^4^. Likewise, the current study found that increases in in-flight deadlift training volume identified astronauts who recovered tibia BMD. Since cramped quarters will be a limiting factor on future exploration-class missions, exercise equipment will need to be optimized for a smaller footprint. Resistance exercise training (particularly deadlifts and other lower-body exercises) will remain a mainstay for mitigating bone loss; however, adding a jumping exercise to on-orbit regimens may further prevent bone loss and reduce daily exercise time. Jumping provides short bouts of high-impact, dynamic loads that promote osteogenesis^20^. The Cologne RSL bedrest study recently demonstrated the effectiveness of three minutes of jumping per training session (using a sledge jump system) for preserving tibia total BMD (via pQCT), muscle mass and strength, and aerobic capacity over 60 days of bed rest^21,22^. Successful implementation of high-load jump-training on-orbit will require an exercise device that mitigates forces transferred to the vehicle, along with an exercise regimen that accounts for astronaut deconditioning.

Aside from resistance exercise, astronauts whose bone did not recover had greater bone turnover at all time points compared with astronauts who recovered^7^. Thus, pre-flight measures of bone resorption and formation may identify astronauts at greatest risk of bone loss who would most benefit from additional countermeasures, be it enhanced exercise or a pharmaceutical intervention. Recent data suggest anti-resorptives in addition to in-flight exercise is more effective for mitigating bone loss than exercise alone^23^. Bone resorption was suppressed and bone loss reduced in astronauts prescribed a bisphosphonate (alendronate) compared with astronauts not on a bisphosphonate^23^. However, alendronate can cause side effects that may not be well tolerated on-orbit (e.g., gastrointestinal issues). Thus, future studies should evaluate the effectiveness and suitability of a better-tolerated anti-resorptive, such as a one-time infusion of zoledronic acid, which prevents bone loss after spinal cord injury^24^.

This study has several limitations. Our small sample size reflects the nature of space health research. Future investigations should include more female astronauts so that sex-differences in bone loss and recovery can be evaluated. While recovery of bone density and strength plateaued between 6 and 12 months after return from space, longer follow-up is needed to confirm whether bone can recover beyond 1-year. Further, although in-flight resistance training is automatically logged, similar records are not available for pre- and post-flight exercise training. Thus, a device-based measure of physical activity and/or ground reaction forces would help confirm exercise findings and tailor in-flight training.

Our findings indicate that microgravity induces irreversible damage to bone strength, density, and trabecular bone microarchitecture. While bone partially recovers after spaceflight, sustained losses represent at least a decade of normal age-related bone loss, potentially advancing onset of osteoporosis. Inter-individual differences in bone’s response to microgravity and recovery after return to Earth are largely explained by mission duration. Unless countermeasures improve, incomplete recovery of bone structure and strength may worsen as missions get even longer. Future work is needed to clarify the temporality of bone loss in space and to optimize countermeasures for mitigating bone loss on long-duration flights. Until then, elevated biomarkers of bone turnover appear to identify astronauts at greatest risk of irreversible bone loss; thus, these individuals may benefit most from enhanced preventative measures.

## Methods

### Study participants

This prospective study included 17 astronauts from the National Aeronautics and Space Administration (NASA), Canadian Space Agency (CSA), European Space Agency (ESA), and Japan Aerospace Exploration Agency (JAXA) who were selected for missions to the International Space Station (ISS)^4^. No astronauts were prescribed anti-resorptives or other bone-related medication before flight and females were pre-menopausal. Astronauts were provided 800 IU vitamin D_3_ supplements daily during flight. This study was approved by the University of Calgary Conjoint Health Research Ethics Board (REB14-0573), NASA Institutional Review Board (NASA7116301606HR), Human Research Multilateral Review Board, and JAXA Institutional Review Board for Human Research. All participants provided written informed consent and all experiments were performed in accordance with relevant guidelines and regulations. We previously reported HR-pQCT and bone biochemistry results at pre-flight and immediately after return from spaceflight in relation to exercise^4^.

### Outcomes

#### HR-pQCT bone microarchitecture, density, and strength

We assessed bone microarchitecture, density, and strength at the bilateral distal radius and tibia before and after spaceflight (pre-flight, return (R) + 0 months, R + 6 months and R + 12 months) using HR-pQCT (XtremeCT II, Scanco Medical, Switzerland; 60.7 μm) with the standard in vivo scanning protocol and advanced cortical analysis^4,9,25^. All HR-pQCT scans were performed at the NASA Johnson Space Center except for one astronaut whose scans were all performed at the VieCuri Medical Centre, Venlo, The Netherlands.

We performed image analysis according to the manufacturer’s standard patient protocol^9,25^ and all contours were visually inspected and manually corrected^26^. We performed 3D image registration to ensure the same bone volume was assessed at each time point^27^. Percent overlap averaged 95% at the tibia and 90% at the radius. Morphological standard measures included total BMD (Tt.BMD; mgHA/cm^3^) and trabecular BMD (Tb.BMD; mgHA/cm^3^), bone volume fraction (Tb.BV/TV; %), number (Tb.N; 1/mm), thickness (Tb.Th; mm), separation (Tb.Sp; mm), connectivity density (Conn.D; 1/mm^3^)^28^, and structure model index (SMI; 0 for plate-like and 3 for rod-like structures)^29^; cortical BMD (Ct.BMD; mgHA/cm^3^), thickness (Ct.Th, mm) and porosity (Ct.Po; %). Reproducibility in our laboratory ranges from < 3% for density, trabecular, and cortical microarchitecture to < 14% for Ct.Po^10^. Root mean squared coefficient of variation (CV_rms_) for Conn.D was 4.1% at the tibia and 6.9% at the radius and CV_rms_ for SMI was 11.3% at the tibia and 10.3% at the radius. Least significant change (LSC) was calculated as (CV_rms_) multiplied by 2.77^30^.

Failure load (F.Load; N) was estimated on the unregistered, segmented HR-pQCT images using custom finite element analysis (μFE) software (FAIM, version 8.0, Numerics88 Solutions Ltd, Canada)^31^. An axial compression test was simulated on each segmented image using a 1% strain, Young’s modulus of 8748 MPa and a Poisson’s ratio of 0.3^32,33^. F.Load CV_rms_ is 1.6% at the tibia and 2.5% at the radius in our laboratory.

After manually scoring motion artifacts on a scale from 1 (no motion) to 5 (discontinuities and significant blurring of the periosteal surface)^34^, we excluded HR-pQCT radius scans from one astronaut with motion > 3 who also did not complete the R + 12 follow-up scans, and the dominant tibia scans from one astronaut due to a previous ankle fracture. We analysed one tibia and radius for each astronaut at each time point based on the side demonstrating the greatest change in Tt.BMD after return from spaceflight (n = 17 for data at tibia and n = 16 at the radius)^4^.

#### Densitometry

Areal bone mineral density (aBMD, g/cm^2^) of the femoral neck (FN), total hip (TH), and lumbar spine (LS) and total, lean, and fat body mass (g) were acquired before and after (R + 0 months, R + 6 months, and R + 12 months) spaceflight using dual X-ray absorptiometry (DXA; Hologic QDR Discovery). Scans of both hips and lumbar spine were conducted at NASA Johnson Space Center in the Bone and Mineral Laboratory and for one crew member at ESA’s DLR Institute of Aerospace Medicine. Scans were analyzed using Hologic’s automated software as previously described^23^. Weekly and day-of test calibration was conducted using a calibration phantom. Precision values at JSC are < 1% for aBMD and body mass^23^.

#### Bone biochemistry

Biochemical data were obtained through data sharing with NASA’s Biochemical Profile and Spaceflight Standard Measures studies. Blood and urine samples were collected before flight at approximately launch (L)-180 and L-45 days, in-flight at approximately flight day (FD)15, FD30, FD60, FD120, and FD180, and upon return (R) at approximately R + 0 months, R + 1 month, R + 6 months, and R + 12 months. Pre-and post-flight urine collections included two consecutive 24-h urine pools while in-flight urine collections were limited to one 24-h urine pool given crew constraints. Blood samples were collected following an overnight fast. Biochemical assays were performed as previously described^4^. Urine and blood biomarkers were analyzed by the NASA Nutritional Biochemistry Lab at JSC. Biomarkers included urine and serum creatinine, urine N-telopeptide (NTx) and C-telopeptide (CTx), urine and serum calcium, osteocalcin, bone-specific alkaline phosphatase (BSAP), sclerostin, 1,25-dihydroxyvitamin D, 25-hydroxyvitamin D, parathyroid hormone, osteoprotegerin (OPG), receptor activator of nuclear factor kappa-Β ligand (RANKL), and procollagen 1 intact N-terminal propeptide (P1NP). For this study, pre-flight results (180 and 45 days before launch) were averaged. Three astronauts did not participate in a collaborative study analyzing CTx; thus, maximum biomarker sample size was n = 17 except for CTx (n = 14).

#### Exercise

Pre- and post-flight exercise was estimated using a health history questionnaire regarding mean frequency and duration of running and cycling sessions. Questions about resistance training included mean sets and repetitions per week for various exercises including squats, deadlifts, and heel raises. Data from the in-flight treadmill and ergometer included mean frequency and duration of sessions, while data from three lower body ARED exercises: squats (back, single leg and sumo), deadlifts (stiff leg, Romanian and sumo), and heel raises (double and single leg) included mean number of sets and repetitions per session. Although recovery programs are not identical across agencies during post-flight recovery, NASA astronauts complete a 45-day reconditioning period supervised by Astronaut Strength and Conditioning Specialists.

### Statistical analysis

All analyses were conducted in Stata (version 16, StataCorp, College Station, USA) using absolute pre- and post-flight values; figures and tables present percent change relative to pre-flight. Changes in bone variables across time (pre-flight, R + 0, R + 6, R + 12) were assessed using mixed effects models with Kenward–Roger small sample size adjustment^35^. Models included fixed effects of time, with a random intercept to allow individuals their own intercept for the effect of time. To examine predictors of bone loss and recovery, additional models included the fixed effects of mission duration dichotomized (< 6 or > 6 months) and interactions with time. Significant interactions were probed using contrasts with small effects.

We used the same side (dominant or non-dominant) hip as the tibia to compare bone recovery measured by DXA to recovery measured by HR-pQCT. Bone was considered recovered if Tt.BMD by HR-pQCT was within the LSC at 12-month follow-up. Mixed effects models examined change in biochemical markers of bone turnover across time (pre-flight, FD15, FD30, FD60, FD120, FD180, and post-flight at R + 0, R + 1, R + 6, and R + 12 months). Bonferroni correction accounted for multiple comparisons. We log-transformed BSAP, osteocalcin, P1NP, sclerostin, 25-hydroxy vitamin D, and RANKL for analyses, but present raw data in tables. We excluded one outlying RANKL measure that was five standard deviations above the mean. Models additionally included the fixed effect of bone recovery status (complete or incomplete recovery of Tt.BMD at R + 12) to evaluate to skeletal recovery relative to change in biomarkers. Change in exercise volume over time (pre-flight, in-flight, R + 6, R + 12) was examined using mixed effects models and included the fixed effect of bone recovery status. Model assumptions were assessed graphically using plots of residuals and significance was set at *p* < 0.05.

## Supplementary Information

Supplementary Information.

## Data Availability

Data are not publicly available due to astronaut privacy concerns but may be made available in unidentifiable format from the corresponding author upon reasonable request.
