# Supplementary material for: Incomplete recovery of bone strength and trabecular microarchitecture at the distal tibia 1 year after return from long duration spaceflight
Source: Sci Rep. 2022 Jun 30;12:9446. doi: 10.1038/s41598-022-13461-1 (PMC9247070; doi:10.1038/s41598-022-13461-1)
Supplement: Supplementary file 1 — Supplementary Information. [file 41598_2022_13461_MOESM1_ESM.pdf]

Supplementary Appendix

Incomplete recovery of bone strength and trabecular microarchitecture at the distal tibia one year after return from long duration spaceflight

Table of Contents

Supplementary Figure S1 3
Supplementary Figure S2 4
Supplementary Figure S3 5
Supplementary Table S1 6

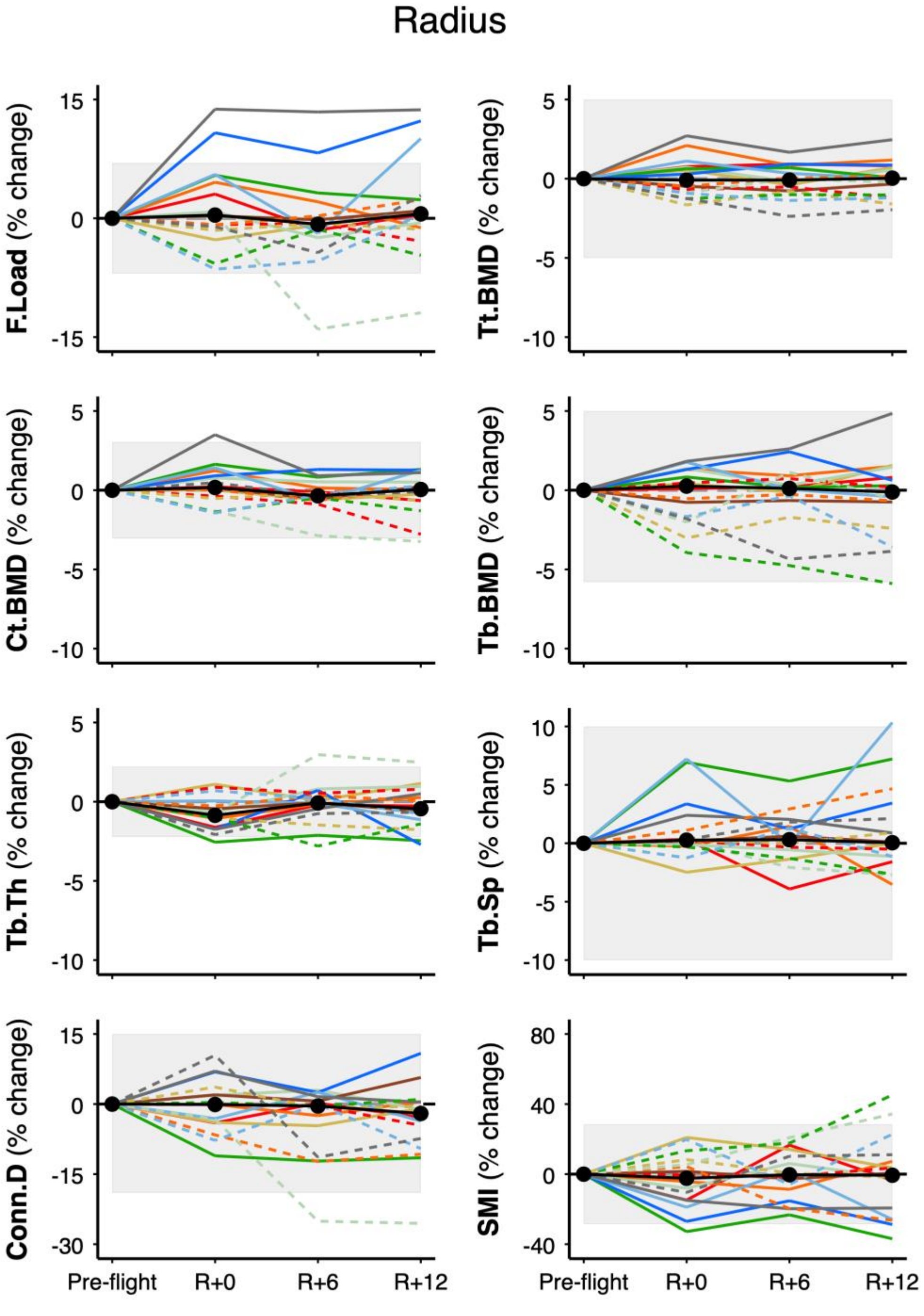

Supplementary Figure S1. **Radius bone strength, density, and trabecular microarchitecture.** Values are percent change from pre-flight at return (R+0), 6-months (R+6), and 12-months (R+12) recovery. Dashed lines for astronauts on > 6-month missions (n=8) and solid lines for astronauts on < 6-month missions (n=9). Black circles connected by thick solid line indicates median group change. Shaded bars indicate least significant change.[15] F.Load, failure load; Tt.BMD, total bone mineral density; Ct.BMD, cortical bone mineral density; Tb.BMD, trabecular bone mineral density; Tb.Th, trabecular thickness; Tb.Sp, trabecular separation; Conn.D, connectivity density; SMI, structure model index.

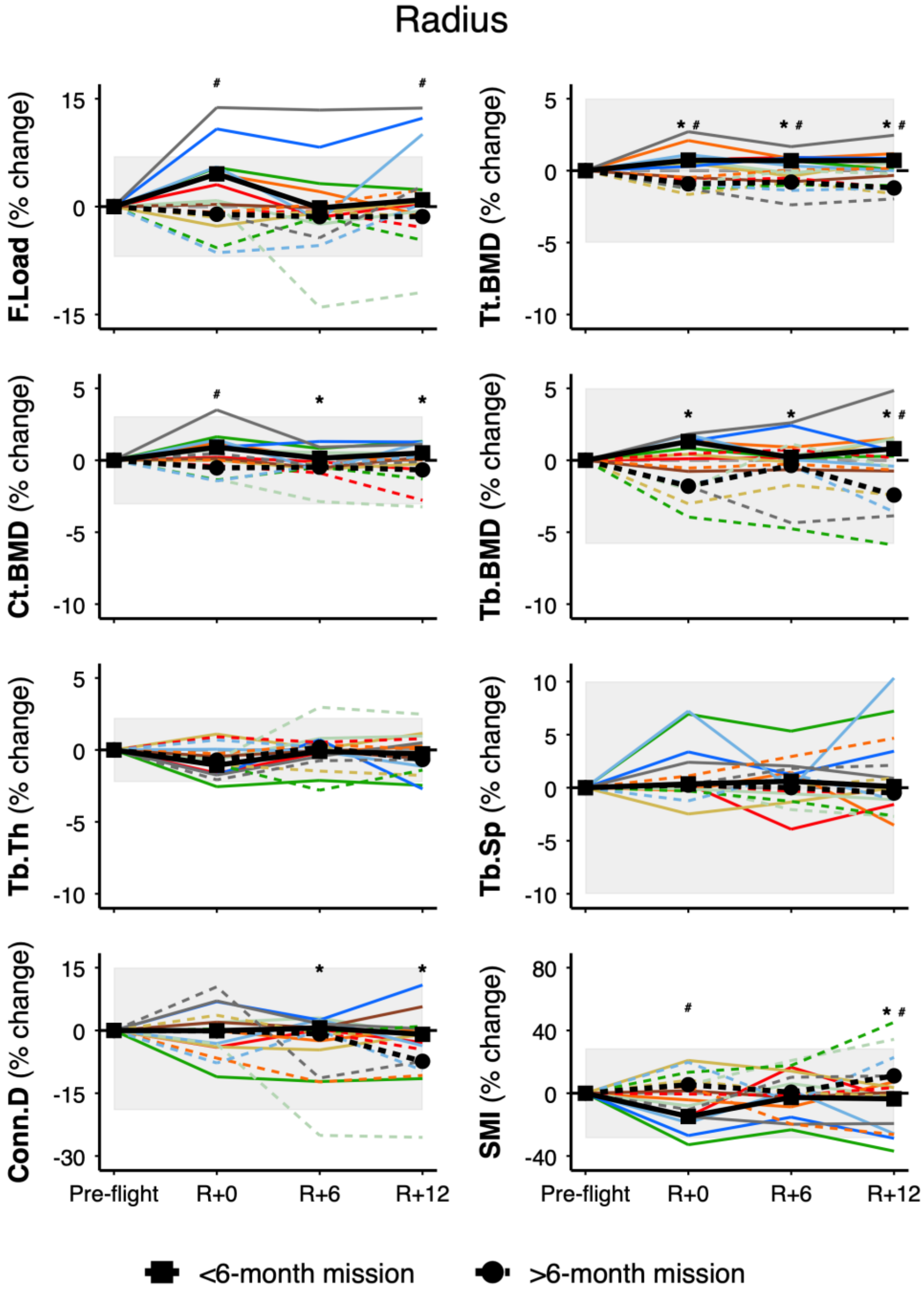

Supplementary Figure S2. Radius bone strength, density, and trabecular microarchitecture by mission duration. Values are percent change from pre-flight at return (R+0), 6-months (R+6), and 12-months (R+12) recovery. Dashed lines for astronauts on > 6-month missions (n=8) and solid lines for astronauts on < 6-month missions (n=9). Black circles connected by thick dashed line indicate median change for astronauts on > 6-month missions and black squares connected by thick solid line indicate median change for astronauts on < 6-month missions. Shaded bars indicate least significant change.[15] F.Load, failure load; Tt.BMD, total bone mineral density; Ct.BMD, cortical bone mineral density; Tb.BMD, trabecular bone mineral density; Tb.Th, trabecular thickness; Tb.Sp, trabecular separation; Conn.D, connectivity density; SMI, structure model index.*$p<0.05$ from pre-flight for astronauts on > 6-month missions; $^{\#}p<0.05$ from pre-flight for astronauts on < 6-month missions.

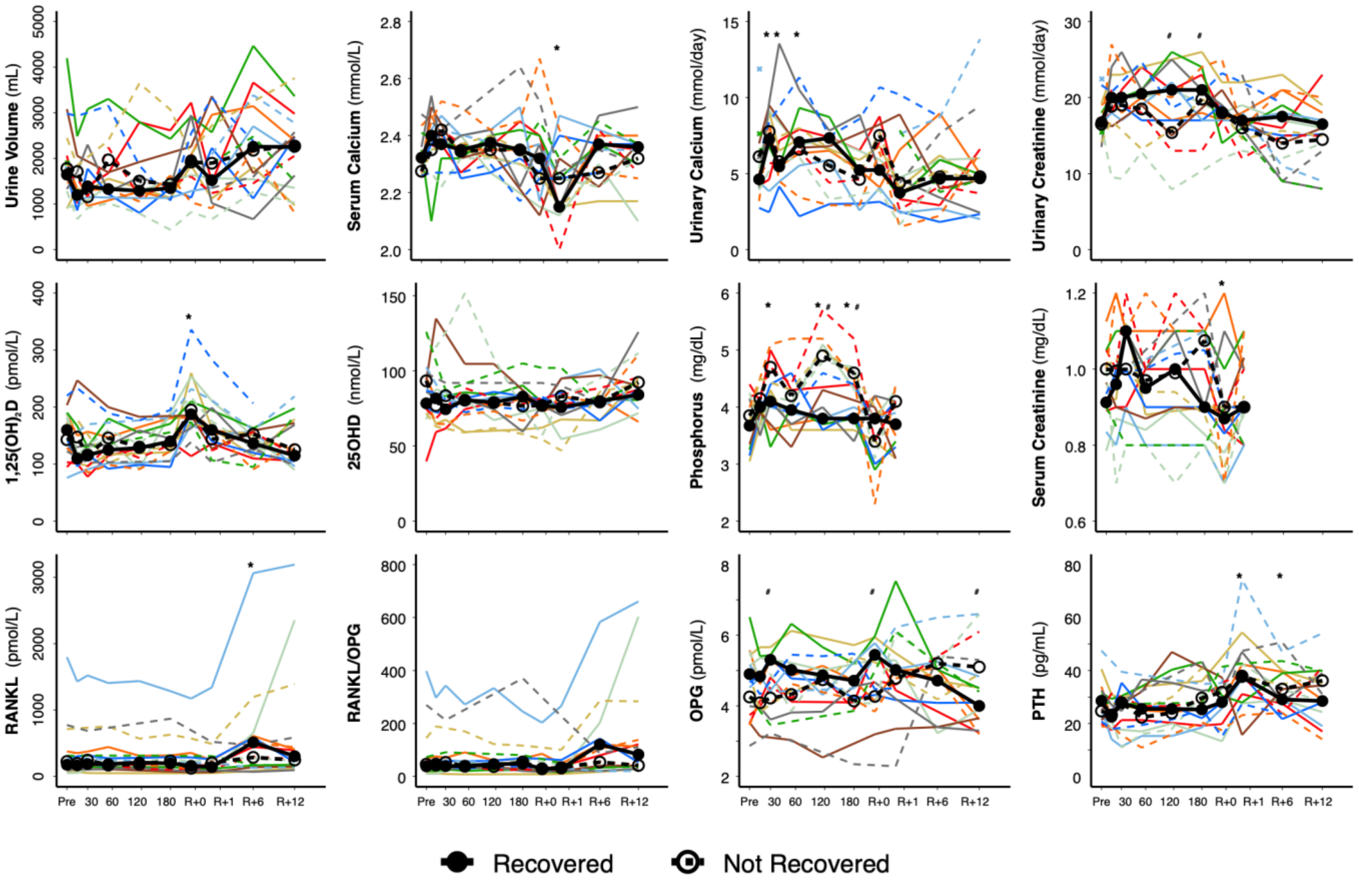

Supplementary Figure S3. Biomarkers of bone turnover relative to bone recovery at pre-flight (Pre), flight days 15, 30, 60, 120, 180, and return (R+0), 1-month (R+1), 6-months (R+6), and 12-months (R+12) recovery. Median group change for astronauts who recovered (n=8) total BMD at R+12 are indicated by black circles connected by a thick solid line, while median group change in astronauts who did not recover (n=9) total BMD at R+12 are indicated by open circles connected by a thick dashed line. Colored dashed lines for astronauts on > 6-month mission and solid lines for astronauts on < 6-month mission. 24-hr urine volume; serum calcium; urinary calcium; urinary creatinine; 1,25(OH)2D, 1,25 di-hydroxy vitamin D; 25OHD, 25-hydroxyvitamin D; phosphorus; serum creatinine; RANKL, RANKL/OPG, PTH, intact parathyroid hormone; OPG, osteoprotegerin. Note: three astronauts on > 6-month missions did not complete all in-flight measures; thus, for urine volume, calcium, and creatinine these astronauts are indicated by an 'x' at pre-flight and trajectory between R+0 and R+12. Phosphorus and serum creatinine were not measured beyond R+1. *$p<0.05$ from pre-flight for entire group. $^{\#}p<0.05$ between astronauts whose tibia total BMD recovered and astronauts whose tibia total BMD did not recover, $p<0.05$.

Supplementary Table S1. Pre-flight biochemical markers of bone turnover and bone metabolism and change from pre-flight.

	Pre-flight	ΔFD15	ΔFD30	ΔFD60	ΔFD120	ΔFD180	ΔR+0m	ΔR+1m	ΔR+6m	ΔR+12m
N[#]	17	14	13	14	13	15	17	17	17	16
**Bone resorption**										
CTx (ug/d)	1513 (1068, 1935)	**1309 (709, 2191)[b]**	**1478 (1155, 2359)[b]**	**2267 (1327, 2831)[b]**	**1296 (798, 2788)[b]**	**1625 (754, 1970)[b]**	**1325 (904, 1848)[b]**	369 (-60, 662)	-2 (-332, 150)	-231 (-400, 150)
CTx (ug/mmol Cr)	91 (63, 135)	**44 (31, 75)[b]**	**80 (38, 111)[b]**	**101 (64, 143)[b]**	**92 (64, 124)[b]**	**82 (49, 115)[b]**	**57 (44, 113)[b]**	20 (-2, 44)	-3 (-24, 11)	-10 (-30, 20)
NTx (nmol/d)	390 (323, 439)	**209 (163, 393)[b]**	**277 (126, 338)[b]**	**357 (174, 482)[b]**	**196 (131, 406)[b]**	**216 (96, 389)[b]**	**251 (136, 309)[b]**	86 (54, 158)	-131 (-141, -32)	-105 (-176, -7)
NTx (nmol/mmol Cr)	23 (20, 28)	**8 (4, 21)[b]**	**9 (5, 14)[b]**	**12 (8, 21)[b]**	**10 (6, 18)[b]**	**12 (5, 22)[b]**	**12 (8, 20)[b]**	4 (2, 10)	-3 (-8, 6)	-3 (-6, 5)
**Bone formation**										
BSAP (U/L)	21.2 (18.0, 22.5)	-1.1 (-2.3, 0.9)	0.7 (-0.2, 1.0)	1.9 (1.3, 3.4)	**3.7 (1.9, 6.6)[b]**	**4.7 (4.0, 11.4)[b]**	**7.3 (3.5, 10.1)[b]**	**8.6 (4.1, 12.2)[b]**	-0.9 (-1.6, 0.5)	**-2.5 (-3.9, -0.6)[b]**
OC (ng/mL)	20.9 (18.3, 23.6)	1.1 (-0.5, 2.8)	2.0 (0.0, 3.9)	**5.5 (3.8, 9.1)[b]**	**10.7 (7.7, 11.9)[b]**	**14.3 (7.4, 16.4)[b]**	**4.2 (1.1, 7.9)[b]**	**11.7 (7.3, 17.9)[b]**	**4.2 (1.0, 8.3)[a]**	0.6 (-3.8, 2.7)
P1NP (ug/L)	48.8 (42.3, 57.1)	-4.5 (-9.0, -0.6)	10.6 (3.8, 18.9)	**26.8 (14.0, 37.5)[b]**	**41.0 (22.5, 52.9)[b]**	**51.3 (38.4, 66.2)[b]**	**40.4 (13.8, 49.4)[b]**	**57.5 (45.5, 71.1)[b]**	7.8 (0.5, 14.1)	-1.4 (-11.3, 9.6)
**Osteocyte activity**										
Sclerostin (pmol/L)	26.2 (21.3, 31.3)	2.5 (-0.9, 4.2)	2.9 (1.2, 9.1)	1.9 (-1.0, 5.6)	2.0 (0.3, 5.1)	1.3 (-1.8, 3.5)	-0.2 (-3.4, 1.1)	-0.3 (-2.2, 1.6)	2.7 (-2.1, 7.3)	2.2 (-0.5, 5.2)
**Regulators of bone metabolism**										
Urinary calcium (mmol/d)	4.86 (4.20, 6.14)	**2.39 (0.41, 3.79)[b]**	**1.36 (0.61, 1.86)[a]**	**1.83 (1.08, 3.62)[b]**	1.44 (-0.18, 2.13)	0.33 (-0.38, 1.71)	0.58 (-0.14, 2.00)	-0.72 (-2.04, 0.19)	-0.68 (-1.28, 0.88)	-0.18 (-1.63, 1.50)
Serum calcium (mmol/L)	2.30 (2.28, 2.35)	0.08 (0.04, 0.12)	0.05 (-0.03, 0.14)	0.04 (-0.03, 0.10)	0.06 (0.01, 0.09)	0.07 (-0.06, 0.12)	-0.01 (-0.07, 0.05)	**-0.10 (-0.17, -0.01)[a]**	0.02 (-0.06, 0.07)	0.01 (-0.06, 0.06)
PTH (pg/mL)	25.2 (22.1, 31.5)	-2.6 (-5.5, 1.4)	-3.2 (-8.0, 1.4)	-0.4 (-7.9, 2.6)	1.3 (-5.6, 4.9)	-0.1 (-4.6, 5.1)	0.3 (-1.3, 5.9)	**12.9 (6.6, 14.7)[b]**	**5.6 (-0.4, 7.6)[a]**	5.9 (-3.9, 10.2)
1,25(OH)$_2$D (pmol/L)	145 (128, 187)	-8 (-49, 13)	-26 (-43, 1)	-25 (-43, 6)	-36 (-45, -2)	-6 (-25, 30)	**36 (17, 85)[b]**	-4 (-20, 63)	-25 (-40, 5)	3 (-50, 21)
25OHD (nmol/L)	86 (76, 96)	-4 (-9, 4)	-7 (-17, 1)	1 (-13, 13)	-7 (-11, 6)	-14 (-21, 4)	-10 (-18, 0)	-10 (-20, 2)	-7 (-14, 4)	3 (-15, 20)
Phosphorus (mg/dL)	3.8 (3.4, 4.0)	0.4 (0.0, 0.7)	**0.3 (-0.1, 1.1)[b]**	0.1 (-0.1, 0.6)	**0.7 (0.3, 1.2)[b]**	**0.5 (0.1, 0.7)[a]**	-0.2 (-0.6, 0.3)	0.0 (-0.1, 0.2)	NA	NA
OPG (pmol/L)	4.7 (3.7, 5.2)	0.1 (-0.8, 0.2)	-0.1 (-0.6, 0.6)	0.1 (-0.5, 0.7)	-0.2 (-0.4, 0.3)	0.2 (-0.7, 0.6)	0.2 (-0.4, 0.7)	0.2 (-0.2, 0.9)	-0.4 (-0.7, 1.3)	-0.1 (-0.8, 0.7)
RANKL (pmol/L)	217 (124, 312)	2 (-3, 27)	4 (-14, 11)	5 (-18, 41)	3 (-42, 19)	-6 (-60, 17)	-20 (-46, -6)	-19 (-59, -5)	**113 (-2, 337)[a]**	42 (-14, 230)
RANKL/OPG	46 (31, 68)	3 (0, 9)	4 (-4, 6)	4 (-10, 14)	1 (-9, 5)	-2 (-10, 7)	-6 (-17, 13)	-11 (-23, -3)	12 (-2, 89)	14 (-3, 76)
**Chemistry**										
24-hr Urine volume (mL)	1741 (1475, 2062)	-588 (-789, 7)	-405 (-674, 128)	-207 (-498, 172)	-490 (-841, 254)	-232 (-832, 191)	-149 (-438, 694)	-15 (-388, 306)	572 (-147, 904)	437 (-334, 830)

Urinary Creatinine (mmol/d)	17 (16, 18)	2 (1, 4)	3 (1, 4)	3 (1, 5)	2 (-3, 5)	3 (-1, 6)	1 (-2, 3)	0 (-1, 0)	-1 (-5, 2)	-1 (-3, 0)
Serum Creatinine (mg/dL)	1.00 (0.90, 1.00)	0.02 (-0.04, 0.08)	0.05 (0.00, 0.10)	-0.03 (-0.05, 0.06)	0.03 (-0.05, 0.10)	0.00 (-0.10, 0.10)	**-0.07 (-0.14, -0.03)[a]**	-0.04 (-0.10, 0.00)	NA	NA

Data are median (interquartile range) for pre-flight and median change from pre-flight.
[a]***p*<0.05; [b]*p*<0.01** compared with pre-flight (mean of L-180 and L-45) based on linear mixed effects model with small sample adjustment and Bonferroni correction. BSAP, OC, P1NP, sclerostin, 25OHD, RANKL, and RANKL/OPG were log-transformed for analysis. [#]maximum sample size for data collection period. Three participants did not have CTx analyzed at any time point, phosphorus was not analyzed at R+6, nor was serum creatinine at R+6 or R+12.
CTx, type I collagen C-terminal cross-linked telopeptide; NTx, type I collagen N-terminal cross-linked telopeptide; BSAP, bone-specific alkaline phosphatase; OC, osteocalcin; P1NP, procollagen type 1 amino-terminal propeptide; PTH, intact parathyroid hormone; 1,25$(OH)_2$D, 1,25 di-hydroxy vitamin D; 25OHD, 25-hydroxyvitamin D; OPG, osteoprotegerin.
